# Reply to Comments on Proteomic Investigations of Two Pakistani *Naja* Snake Venom Species Unravel the Venom Complexity, Posttranslational Modifications, and Presence of Extracellular Vesicles. *Toxins* 2020, *12*, 669

**DOI:** 10.3390/toxins12120781

**Published:** 2020-12-08

**Authors:** Aisha Munawar, Benjamin Dreyer, Hartmut Schlüter, Christian Betzel

**Affiliations:** 1 Department of Chemistry, Faculty of Natural Sciences, University of Engineering and Technology, Lahore 54890, Pakistan; 2 Institute of Clinical Chemistry and Laboratory Medicine, Mass Spectrometric Proteomics, University Medical Centre Hamburg-Eppendorf (UKE), Martinistraße 52, 20246 Hamburg, Germany; b.dreyer@uke.de (B.D.); hschluet@uke.de (H.S.); 3 Laboratory for Structural Biology of Infection and Inflammation, Institute of Biochemistry and Molecular Biology, Deutsches Elektronen-Synchrotron, University of Hamburg, Building 22a, Notkestr.85, 22603 Hamburg, Germany; christian.betzel@uni-hamburg.de

We appreciate the commentary on our article, and we would like to take the opportunity to address several points raised in the reviewers’ commentary.

In the comments, it is stated “… it [the study] does not provide information on the abundance of each protein family in the venoms of the snakes”.

This is of course not true, because we applied a state-of-the-art analysis of the proteome of the two snakes, which is much more precise than “the most common method”, using liquid chromatography (LC) for fractionation of the venom and subsequent SDS-PAGE of the individual fractions. From the viewpoint of the basics of analytical chemistry, such an approach is only suitable for quantification (which is nothing else than the determination of abundance), if internal standards (in the best case, venom proteins labeled with stable isotopes) are added. In the case of the approach combining LC with SDS-PAGE (sodium dodecyl sulfate polyacrylamide gel electrophoresis), without applying internal standards, only extremely rough conclusions can be drawn regarding the quantities of proteins for the following reasons:

- Every separation step underlies the law of mass action, thereby multiplying the error with the number of fractionation steps. Because of this problem, in analytical chemistry internal standards are used;
- A single Coomassie stained band does contain much more than one protein. With modern methods of proteomics, we usually detect dozens, very often hundreds of proteins in one single SDS-PAGE band. Even in spots of two-dimensional electrophoresis, we identify on average more than 100 proteins.

With our proteomics approach, we directly digested the proteins in the venom, and injected the resulting peptides thereafter directly into the mass spectrometric system. Thereby, we excluded by a large degree the systematic error induced by the law of mass action.

For estimating the abundance of the proteins, the number of identified peptides per protein was used in this study. This indeed gives a rough classification of the abundance (although the error is not high) of the proteins in the venom, which in our opinion is appropriate, since our main focus was on the identification of proteins in the venom and not on the accurate quantification. Our study focused on the data generated with a refinement approach of database searching and additional de novo peptide sequencing of unidentified MS/MS spectra. This approach aimed for a deep characterization of the studied venoms, since no sufficient protein sequence database was available for the two *Naja* species. The mass spectrometric data was searched against a collection of protein sequences derived from gene sequences of different snake species under the taxonomy “Serpentes”. Identified peptides were grouped into proteins, and similar proteins are then assembled into protein groups, e.g., the same protein from different species. Furthermore, we uploaded all the data from the mass spectrometric analysis in the PRIDE database [1] (with the dataset identifier PXD018726 and 10.6019/PXD018726), so that everybody can reprocess the data at any time. Thus, a view on the spectral counts is possible, which will increase the accuracy of the quantification. This can be performed by reprocessing the data performing spectral counting of the peptide spectra matches (PSMs) of each protein group. For the convenience of the readers of the journal *Toxins*, we can add a table about the relative quantities of the peptides based on spectral counting data, if desired. The number of the PSMs for one protein group can be taken as a measure for the abundance, since more abundant peptides will be measured at a higher frequency in the mass spectrometer than less abundant ones. The protein identification data for the search against “sperpentes” is included in the deposited mass spectrometic data under the identifier PXD018726 at ProteomeXchange.

A further aspect, which is addressed in the comments, is the question about the sum of the toxic action of the identified venom proteins in the venoms of the two snakes.

The authors of this comment recommended answering this question under the aspect that the abundance of the individual proteins is the main criterion.

We do not agree for the following reasons:

(1) The absolute pharmacological action of a single venom depends not only on the absolute quantity, but also on its potency (with respect to its enzymatic activity);
(2) The potency of a protein in the venom depends on its exact composition of atoms, and of course the three-dimensional fold. Already, small changes in the composition of atoms and the sequence and location of amino acids can drastically change the action and alter the potency of the function of a protein (please refer to the respective publications about protein species, proteoforms (synonyms), e.g., references [2,3,4,5].

In the case of a mixture of many different active components of a venom, synergic effects are also common and need to be considered. An estimation of the overall toxic action thereby is critical.

In summary, the data, which we provided in our study and corresponding manuscript [6], are much more precise than many other, older published investigations not using the latest proteomics. However, the conclusion that can be drawn regarding the estimation of the overall toxic effects still is, of course, also approximated in our study.

Regarding the criticism about the terms “complexity” and “diversity”, we cannot really follow this comment, because both terms were used only once and in these cases have not been mixed with the meaning of “amounts” and “abundance”.

Similarly, we checked all sentences including the terms “amounts” and “abundance”. In no case were these terms mixed with the terms “complexity” and “diversity”.

Finally, as is good practice for the journal *Toxins*, and as practiced for other comments sent in before, the person or group sending the comment should identify themselves, and not stay anonymous.
